# Engaging Traditional Medicine Providers in Colorectal Cancer Screening Education in a Chinese American Community: A Pilot Study

**DOI:** 10.5888/pcd11.140341

**Published:** 2014-12-11

**Authors:** Jun Wang, Adam Burke, Janice Y. Tsoh, Gem M. Le, Susan Stewart, Ginny Gildengorin, Ching Wong, Elaine Chow, Kent Woo, Tung T. Nguyen

**Affiliations:** Author Affiliations: Adam Burke, San Francisco State University, Asian American Research Center on Health, San Francisco, California; Janice Y. Tsoh, University of California and Asian American Research Center on Health, San Francisco, California; Gem M. Le, Ginny Gildengorin, Ching Wong, University of California, San Francisco, California; Susan Stewart, University of California, Davis, California; Elaine Chow, NICOS Chinese Health Coalition, San Francisco, California; Kent Woo, NICOS Chinese Health Coalition and Asian American Research Center on Health, San Francisco, California; Tung T. Nguyen, University of California and Asian American Research Center on Health, San Francisco, California.

## Abstract

**Introduction:**

Although colorectal cancer (CRC) screening is effective in preventing colon cancer, it remains underused by Asian Americans. Because Chinese Americans often use traditional Chinese medicine (TCM), we conducted a pilot study to explore the feasibility and acceptability of having TCM providers deliver education about CRC screening.

**Methods:**

Four TCM providers (2 herbalists and 2 acupuncturists) were trained to deliver small-group educational sessions to promote CRC screening. Each provider recruited 15 participants aged 50 to 75. Participants completed a baseline survey on CRC-related knowledge, attitudes, and behaviors and then attended one 2-hour educational session delivered by the providers in Cantonese or Mandarin. Three months later, participants completed a postintervention survey.

**Results:**

Sixty participants were recruited from the San Francisco Chinatown neighborhood. The average age was 62.4 years. Most participants had limited English proficiency (96.7%), annual household income less than $20,000 per year (60%), and low educational attainment (65.1% < high school education). At postintervention (n = 57), significant increases were found in having heard of CRC (from 52.6% to 79.0%, *P* < .001) and colon polyps (from 64.9% to 84.2%, *P* < .001). Knowledge regarding screening frequency recommendations also increased significantly. The rate of ever having received any CRC screening test increased from 71.9% to 82.5% (*P* <.001). The rate of up-to-date screening increased from 70.2% to 79.0% (*P* = .04).

**Conclusion:**

The findings suggest that TCM providers can be trained to deliver culturally and linguistically appropriate outreach on CRC screening within their community. Participants reached by TCM providers increased CRC knowledge and self-reported CRC screening.

## Introduction

Colorectal cancer (CRC) is the third most commonly diagnosed cancer among both Chinese American men and women (1). CRC screening is a cost-effective approach to reduce CRC mortality, but CRC screening rates remain low among Asian Americans (2). Analysis of the 2007 California Health Interview Survey (CHIS) revealed that Asian Americans with low health literacy and limited English proficiency were most vulnerable to CRC screening disparities (3). A possible barrier to CRC screening among Chinese Americans may be lack of awareness (4). Other studies of Chinese immigrants have noted a lack of understanding of CRC screening as a preventive measure, as well as challenges with language, transportation, access to health care, limited social support for use of screening services, and lack of health insurance (5–7). A review of 30 culturally appropriate cancer screening intervention studies found that community-based and lay health worker strategies were effective in improving cancer screening rates among Asian Americans. Commonly used communication channels were churches and Asian grocery stores (8).

Traditional Chinese medicine (TCM) providers may provide another useful community-based channel to convey culturally appropriate information about CRC screening. Use of TCM, such as acupuncture and herbal medicine, is high among Chinese Americans. The 2002 National Health Interview Survey (NHIS) found that Asian American women were significantly more likely to use acupuncture compared with other racial/ethnic groups (9). Other national surveys have also found that use of acupuncture, herbs, and related services is high among Asian Americans (10–12). Traditional medicine providers have health message credibility, are readily accessible, and embody the cultural and linguistic characteristics of the communities they serve. As such, they have the potential to be an effective channel for health promotion. International studies have shown the practical value of integrating traditional healers into community health messaging, including studies finding positive outcomes in HIV care, oral rehydration therapy, water treatment, sanitation and hygiene, nutrition education, and birthing practices (13–15). Two studies conducted in the United States found that traditional Chinese medicine providers are interested in participating in community-based preventive education programs (16,17).

To date, no published studies have tested a community-based cancer screening intervention using traditional healers in the United States. To fill this gap, we conducted a pilot study during 2008–2013 to explore the feasibility and acceptability of TCM providers offering education in CRC prevention in the Chinatown neighborhood of San Francisco. The hypothesis was that educational sessions delivered by TCM providers would increase participants’ CRC screening knowledge and screening rates.

## Methods

**TCM provider recruitment.** The institutional review boards at San Francisco State University and University of California, San Francisco, approved all research protocols. TCM providers were identified through local TCM professional associations and providers’ networks. The providers were expected to 1) complete a 4-hour training; 2) recruit 15 participants from among their clients and social networks, including at least 8 who had never received CRC screening; 3) conduct a 90-minute small-group education session in either Cantonese or Mandarin with participants; and 4) participate in a final exit interview. Providers received $1,000 as compensation for their participation for spending approximately 20 hours in training, recruiting, conducting educational sessions, and in postintervention feedback interviews.

Among the 10 Chinatown area TCM providers approached, 3 herbalists refused to participate because of concerns of distressing their clients. One acupuncturist could not participate because of a time conflict. Two acupuncturists were not selected because they had a small percentage (less than 30%) of Chinese clients. Four TCM providers (2 female acupuncturists and 2 male herbalists) were selected on the basis of their interest in participating and suitable work schedules. All had graduated from TCM colleges in Canton or Hong Kong, had practiced in San Francisco for more than 10 years, and served a range of 50 to 100 clients per week. With 6 of 10 TCM providers being willing to participate, the possible participation rate of 60% in this study is similar to rates of participation by Western health professionals in a postal survey (59%) (18) and electronic health record research (42%) (19), and is much higher than the 1% to 2% rates in typical intervention studies (20).

**TCM provider materials and training.** TCM providers received 4 hours of training (2 hours each on 2 consecutive days) in Mandarin or Cantonese. A training manual was created with the following content. On the first day, providers learned about the project goals and structure, recruitment strategies, and how to use an integrative CRC screening education flipchart. The 38-page flipchart was based on a previously developed and tested biomedical instrument used in a larger study funded by the National Cancer Institute (NCI) (NCI R01CA138778). The revised flipchart was modified to include culturally appropriate information on healthy lifestyle, colorectal cancer, and CRC screening from a perspective that integrates TCM and Western medicine. On the second day, providers were taught about CRC prevention and motivational strategies. The development and pretesting of the educational materials used in this intervention, including the training manual and integrative flipchart, have been reported previously (17,21).

**Participant recruitment.** Each TCM provider recruited 15 participants, which yielded a study sample of 60 participants. Eligibility criteria for participants included self-identifying as Chinese and being aged 50 to 75, available for 2 meetings, and able to stay in the study for 3 months. Each participant received $50 on completion. Following a social-network recruitment strategy, we asked providers to recruit from among their clients as well as personal and professional social networks. Social network-based recruitment is an effective and culturally acceptable strategy with harder to reach groups — including our targeted sample of older Chinese American immigrants with limited-English proficiency — as well as with other Asian Americans groups (8,17,21).

Providers recruited participants using multiple methods, ranging from word-of-mouth to posting flyers about the study at their herb store counter or acupuncture clinic waiting area. Providers were encouraged to recruit unscreened participants, but screened participants were acceptable. Screening rates are fairly high in California (although lower for Asians than for whites) and a mixed cohort is more representative of the actual population that the providers typically serve and meet on a day-to-day basis.

Each participant attended 1 small-group session with their TCM provider. A total of 8 small-group sessions were held with 5 to 8 attendees per group. Most meetings were conducted in the providers’ offices or in other public settings, such as a community organization office space. Participants completed a self-administered preintervention survey before their small-group session. After the survey was completed, the TCM provider convened the group and taught the participants about CRC prevention in Cantonese or Mandarin using the flipchart, followed by a group discussion. The small-group session usually lasted about 90 minutes. The key concepts presented in the sessions focused on TCM and Western medicine health information related to CRC causes and prevention, information on CRC screening, and CRC screening method/frequency recommendations. Approximately 3 months after the session, each participant completed an in-person postintervention survey conducted by a research staff member.

We based the survey instrument on a modified version of an instrument used in a Chinese lay health worker CRC prevention outreach project conducted by the same team (21). The survey included information on participant demographics, CRC knowledge, attitudes, and behaviors, and traditional medicine beliefs and practices. Descriptive statistics included frequency distributions for categorical data and means and standard deviations for continuous data. We evaluated preintervention and postintervention changes in proportions by using generalized estimating equations (GEE) to account for clustering of participants by TCM provider and within-person correlation over time. A bias-corrected sandwich estimator was used because of the small number of clusters. A significance level of .05 (2-sided) was used for all statistical tests. The statistical analysis was performed using SAS version 9.3 (SAS Institute, Inc).

## Results

Each TCM provider recruited 15 eligible participants (N = 60). Participants were 31 (52%) TCM clients, 20 (33%) friends of the providers or the participants, and 9 (15%) other TCM providers. Three participants did not complete the postintervention survey because of illness or travel, for a retention rate of 95%. The mean age of participants was 62.4 years (SD, 6.5) and 61.7% were female (Table 1). Most participants were born in China (93.3%) with a mean length of US residency of 16.8 years. Most reported limited English proficiency (96.7%), with 90% speaking Cantonese and 10% speaking Mandarin at home. Nearly two-thirds had an annual household income less than $20,000 and 65.1% had less than a high school education. Only 5% reported their health as being very good or excellent. Most had health insurance, a place for Western health care, and had seen a Western medical doctor in the previous 12 months. Although demographic data specific to Chinese Americans aged 50 to 75 are unavailable, the sociodemographics of the participants are similar to those of San Francisco Chinatown area residents (22).

**Table 1:** Characteristics of Study Participants at Baseline (n = 60)

Variables	n (%)
**Sociodemographic**	
Age: Mean (SD)	62.4 (6.5)
Sex: Female	37 (61.7)
**Birthplace**	
China	56 (93.3)
US/Other	4 (6.7)
Years in US: Mean (SD)	16.8 (12.8)
**English fluency**	
Fluently/Well	2 (3.3)
So-so/poorly/not at all	58 (96.7)
**Highest level of education**	
6th grade or less	14 (23.4)
7th–11th grade	25 (41.7)
High school diploma/GED	14 (23.3)
Some college or higher	7 (11.6)
**Employment**	
Employed	26 (43.3)
Retired	17 (28.3)
Others	17 (28.3)
Marital status: married	43 (71.7)
**Annual household income**	
Less than $10,000	22 (36.7)
$10,000 to less than $20,000	14 (23.3)
$20,000 or more	22 (26.7)
Don’t know	2 (3.3)
**Health status and health care**	
Self-reported health	
Excellent/very good	3 (5.0)
Good	23 (38.3)
Fair	30 (50.0)
Poor	4 (6.7)
Has family history of colon cancer	2 (3.3)
Has health insurance	53 (88.3)
Has a regular place for Western health care	48 (80.0)
Saw a Western doctor in last 12 months	47 (78.3)

Abbreviations: SD, standard deviation; GED, general education development.

### Changes in knowledge, attitudes, and behaviors

We analyzed the changes in CRC knowledge, beliefs, attitudes, and screening behaviors among the 57 participants who completed both preintervention and postintervention surveys (Table 2). After accounting for clustering of participants by provider, we found significant increases in ever having heard of CRC (from 52.6% to 79.0%, *P* < .001) and of colon polyps (from 64.9% to 84.2%, *P* < .001). Knowledge of CRC screening tests increased for having heard of any test (from 80.7% to 93.0%, *P* < .001) and for colonoscopy and sigmoidoscopy, but not for fecal occult blood test (FOBT). Correct understanding that CRC screening should start at age 50 increased from 54.3% to 66.7% (*P* < .001). Correct understanding of screening intervals (annual) also increased substantially for FOBT (from 36.8% to 59.7%, *P* < .001), sigmoidoscopy (every 5 years) (from 24.6% to 42.1%, *P* < .001), and colonoscopy (every 10 years) (from 14.0% to 40.4%, *P* < .001).

**Table 2:** Colorectal Cancer Knowledge, Beliefs, Attitudes, and Screening Behaviors Among Chinese Participants in a Traditional Chinese Medicine Outreach Program (n = 57)

Variables	Preintervention, n (%)	Postintervention, n (%)	*P* Value^a^
**Knowledge**			
Ever heard of colon cancer	30 (52.6)	45 (79.0)	<.001
Ever heard of colon polyp	37 (64.9)	48 (84.2)	<.001
Ever heard of FOBT	37 (64.9)	39 (68.4)	.58
Ever heard of sigmoidoscopy	35 (61.4)	43 (75.4)	.05
Ever heard of colonoscopy	39 (68.4)	48 (84.2)	<.001
Ever heard of FOBT, sigmoidoscopy, or colonoscopy	46 (80.7)	53 (93.0)	<.001
Colorectal cancer screening should start at age 50	31 (54.3)	38 (66.7)	<.001
FOBT should be done yearly	21 (36.8)	34 (59.7)	<.001
Sigmoidoscopy should be done every 5 years	14 (24.6)	24 (42.1)	<.001
Colonoscopy should be done every 10 years	8 (14.0)	23 (40.4)	<.001
**Beliefs**			
Causes of colorectal cancer			
Fatty diet	47 (82.5)	48 (84.2)	.74
Lack of physical activity	30 (52.6)	33 (57.9)	.17
Alcohol	21 (36.8)	27 (47.4)	.15
Heredity	20 (35.1)	33 (57.9)	.08
Older age	15 (26.3)	16 (28.1)	.73
Colon polyps	37 (64.9)	47 (82.5)	.05
Inflammatory bowel disease	27 (47.4)	29 (50.9)	.38
Negative emotions	18 (31.6)	20 (35.1)	.51
Fate, God’s will	3 (5.3)	5 (8.8)	.65
**How to prevent colorectal cancer**			
Exercise	32 (56.1)	35 (61.4)	.06
Eat more fiber and vegetables	45 (79.0)	49 (86.0)	.29
Take aspirin	6 (10.5)	13 (22.8)	.14
Get medical tests to find blood or colon polyps	45 (79.0)	41 (71.9)	.15
Have regular bowel movements	44 (77.2)	50 (87.7)	.20
See a traditional Chinese healer	13 (22.8)	16 (28.1)	.52
Take traditional Chinese herbs	19 (33.3)	21 (36.8)	.62
Keep a positive attitude	31 (54.4)	24 (42.1)	.11
**Attitudes**			
CRC screening is an important thing to do	56 (98.3)	54 (94.7)	.06
Completing CRC screening is easy to do	42 (73.7)	40 (70.2)	.51
Finding time to complete CRC screening is difficult	28 (50.0)	21 (37.5)	.13
CRC screening may be physically uncomfortable	33 (57.9)	26 (45.6)	.04
Intend to complete CRC screening	44 (77.2)	48 (84.2)	.11
**Colon cancer screening status**			
Ever had FOBT	38 (66.7)	45 (79.0)	<.001
Ever had sigmoidoscopy or colonoscopy	16 (28.1)	19 (33.3)	.06
Ever had FOBT, sigmoid, or colonoscopy	41 (71.9)	47 (82.5)	<.001
Up-to-date FOBT, sigmoidoscopy, or colonoscopy^b^	40 (70.2)	45 (79.0)	.043

Abbreviations: FOBT, fecal occult blood test; CRC, colorectal cancer.

a *P* value accounts for clustering by provider.

b The United States Preventive Services Task Force screening guidelines were the basis for determining up-to-date screening status (23).

At pretest, more than half of the participants believed that exercise, eating more fiber and vegetables, getting medical tests to find blood or polyps in the colon, having regular bowel movements, and keeping a positive attitude could prevent CRC. From preintervention to postintervention, we saw no significant changes in these beliefs. In terms of attitudes, the only significant change was a decrease in the attitude that screening may be physically uncomfortable (from 57.9% to 45.6%, *P* = .04).

Regarding CRC screening behavior, the proportion of participants reporting ever having had an FOBT increased significantly, from 66.7% to 79.0% (*P* < .001). Similarly, ever having had any screening method increased significantly, from 71.9% to 82.5% (*P* < .001). The proportion of those who were up-to-date with CRC screening increased from 70.2% to 79.0% (*P* = .04).

## Discussion

We found that recruiting and training TCM providers to deliver culturally and linguistically appropriate education on CRC screening is feasible. TCM providers were able to recruit community participants from their TCM clientele as well as their social and professional networks, supporting the relevance of a social-network recruitment strategy. This finding suggests that TCM providers can reach individuals who are traditionally considered hard to reach — older, immigrant, and low income Chinese Americans, with limited English proficiency. A recent qualitative study observed that older Chinese immigrants used TCM not only as a primary resource for preventive self-care and for managing illness but also as a way to claim their cultural identity and cope with the challenges of being in a foreign country with a different health care system (24).

The results also show that TCM providers may be effective in increasing participants’ knowledge about CRC and increasing participants’ receipt of CRC screening. After a single 2-hour education session, participants’ knowledge about CRC screening increased significantly from preintervention to 3 months postintervention. We found significant changes in their awareness of colon cancer and screening tests and correct understanding of the recommended time intervals for each screening test, an essential component of effective preventive self-care. No significant changes were seen in beliefs about CRC prevention. This may have been because the flipchart TCM content, developed in conjunction with TCM providers and field tested with their clients, was concordant with many of the participants’ cultural beliefs, such as a common belief in the importance of eating more fiber and vegetables, and getting regular exercise. One significant change in attitude was important. At posttest, fewer participants indicated that they thought CRC screening would be physically uncomfortable. This is a constructive change, because fear of testing has been found to be a deterrent to screening (25).

In terms of changes in actual screening status, the TCM intervention led to significant increases in screening rates, especially for FOBT, and for being up to date in FOBT, sigmoidoscopy, or colonoscopy. Ever having had any CRC screening test increased from 71.9% to 82.5% (*P* = .004). A California population-based study of CRC screening rates among 5 Asian ethnic groups found that 74% of Chinese Americans aged 65 or older reported having been screened (26). This finding is in line with the 71.9% in this study sample who reported at pretest having had any type of screening.

After a single education session with a community-based TCM provider, rates for ever having had CRC screening increased by 10.6% and up-to-date CRC screening increased by 8.8%. Other large sample studies have also found that a respected health care provider can increase patient screening behavior. Screening recommendations given by Western medical doctors significantly increased patient CRC screening (27,28). An analysis of the 2009 California Health Interview Survey with 30,857 respondents reported that the strongest predictor for having received CRC screening in the past 5 years was physician recommendation (29). The findings in this study suggest that TCM providers may have a similar effect on screening behavior. When participants were asked if they would get an FOBT if a TCM provider advised them to do so, the proportion stating “definitely yes” or “probably yes” increased significantly from 83.3% at baseline to 100% at postintervention.

This model holds promise as a low-cost, community-based, culturally relevant screening promotion strategy. The providers in this study specifically noted that their increased understanding of public health prevention concepts and practices benefited their clients by providing information from both health care models — TCM and Western medicine. One way to institutionalize a CRC program such as this would be to offer it as a continuing education (CE) course for acupuncturists. In most states acupuncture is a licensed health care profession, requiring regular CE for license renewal. A day-long hepatitis education program conducted for acupuncturists in 3 California cities attracted 1,000 attendees. The event had a modest registration fee and provided 8 California CE units (16). A cost-effective 1-day CE program could be developed and delivered using a flipchart and training manual such as the one developed in this pilot study. This would be easily implemented, provide an excellent model for related public health promotion efforts, bridge providers from diverse systems, extend outreach into ethnic communities, and cost very little.

One limitation of this study is that since the participants were recruited through the TCM providers’ client and personal social networks in 1 city, the sample was not population-based and does not reflect the Chinese American population in general. However, the study does show that TCM providers can access potentially hard-to-reach individuals. Given the small sample size and the goal of examining the feasibility of a CRC screening social network model, no control group was available to account for secular changes in the community. That said, it is unlikely that other factors during this 3-month period are responsible for all of the changes observed in this pilot study, including change in knowledge of screening methods, correct knowledge of screening intervals, and improvements in screening status.

In addition, receipt of CRC screening was based on self-report, and little is known about the accuracy of self-report of CRC screening among Chinese Americans. Providers were paid $1,000 to participate, which may limit generalizability, although once trained, the TCM providers may be willing to conduct the outreach activities for a smaller payment or ideally uncompensated, as part of their general patient practice. As a pilot study, the sample size is not large enough to examine diverse factors that may influence behavior change, such as the sex of the provider. Future studies using a larger sample and including a randomized controlled trial should be conducted to confirm the findings of this pilot study and delineate how provider characteristics may affect the receipt of cancer screening messages.

Incorporating traditional healers into public health promotion programs may be another useful way to improve outreach effectiveness particularly with hard-to-reach populations. This study shows that it is feasible to recruit and train TCM providers to deliver community-based CRC screening. There is value in exploring this model further, as a way to expand the public health safety net by working with culturally capable, respected, community-embedded traditional health providers. A larger randomized controlled trial of this strategy would provide valuable information on the potential for this model of integrative public health promotion and disease prevention.

## References

[1] Gomez SL , Noone AM , Lichtensztajn DY , Gibson JT , Liu L , Morris C , Cancer incidence trends among Asian American populations in the United States, 1990-2008. J Natl Cancer Inst 2013;105(15):1096-110. 23878350 10.1093/jnci/djt157 PMC3735462

[2] Hwang H . Colorectal cancer screening among Asian Americans. Asian Pac J Cancer Prev 2013;14(7):4025–32. 10.7314/APJCP.2013.14.7.4025 23991947

[3] Sentell T , Braun KL , Davis J , Davis T . Colorectal cancer screening: low health literacy and limited English proficiency among Asians and Whites in California. J Health Commun 2013;18(Suppl 1):242–55. 10.1080/10810730.2013.825669 24093359 PMC3815112

[4] Homayoon B , Shahidi NC , Cheung WY . Impact of Asian ethnicity on colorectal cancer screening: a population-based analysis. Am J Clin Oncol 2013;36(2):167–73. 10.1097/COC.0b013e3182439068 22441340

[5] Lin JS , Finlay A , Tu A , Gany FM . Understanding immigrant Chinese Americans’ participation in cancer screening and clinical trials. J Community Health 2005;30(6):451–66. 10.1007/s10900-005-7280-5 16370055

[6] McWhirter JE , Todd LE , Hoffman-Goetz L . Beliefs about causes of colon cancer by English-as-a Second-Language Chinese immigrant women to Canada. J Cancer Educ 2011;26(4):734–9. 10.1007/s13187-011-0258-3 21800045

[7] Le TD , Carney PA , Lee-Lin F , Mori M , Chen Z , Leung H , Differences in knowledge, attitudes, beliefs, and perceived risks regarding colorectal cancer screening among Chinese, Korean, and Vietnamese sub-groups. J Community Health 2014;39(2):248–65. 10.1007/s10900-013-9776-8 24142376 PMC3943615

[8] Hou SI , Sealy DA , Kabiru CW . Closing the disparity gap: cancer screening interventions among Asians — a systematic literature review. Asian Pac J Cancer Prev 2011;12(11):3133–9. 22394003

[9] Burke A , Upchurch D , Dye C , Chyu L . Acupuncture use in the United States: findings from the National Health Interview Survey. J Altern Complement Med 2006;12(7):639–48. 10.1089/acm.2006.12.639 16970534

[10] Ahn AC , Ngo-Metzger Q , Legedza AT , Massagli MP , Clarridge BR , Phillips RS . Complementary and alternative medical therapy use among Chinese and Vietnamese Americans: prevalence, associated factors, and effects of patient-clinician communication. Am J Public Health 2006;96(4):647–53. 10.2105/AJPH.2004.048496 16380575 PMC1470548

[11] Wu AP , Burke A , LeBaron S . Use of traditional medicine by immigrant Chinese patients. Fam Med 2007;39(3):195–200. 17323211

[12] Wade C , Chao MT , Kronenberg F . Medical pluralism of Chinese women living in the United States. J Immigr Minor Health 2007;9(4):255–67. 10.1007/s10903-007-9038-x 17431784

[13] Furin J . The role of traditional healers in community-based HIV care in rural Lesotho. Community Health (Bristol) 2011;36(5):849–56. 10.1007/s10900-011-9385-3 21374087

[14] Hoff W . Traditional healers and community health. World Health Forum 1992;13(2-3):182–7. 1418332

[15] Mills E , Singh S , Wilson K , Peters E , Onia R , Kanfer I . The challenges of involving traditional healers in HIV/AIDS care. Int J STD AIDS 2006;17(6):360–3. 10.1258/095646206777323382 16734953

[16] Chang ET , Lin SY , Sue E , Bergin M , Su J , So SK . Building partnerships with traditional Chinese medicine practitioners to increase hepatitis B awareness and prevention. J Altern Complement Med 2007;13(10):1125–7. 10.1089/acm.2007.0655 18166125

[17] Wang J , Burke A , Tsoh JY , Le GM , Wong C , Chow E , Exploring a culturally relevant model of cancer prevention involving traditional Chinese medicine providers in a Chinese American community. Eur J Intern Med 2014;6(1):21–8. 10.1016/j.eujim.2013.09.008 PMC4374358 25821531

[18] Cook JV , Dickinson HO , Eccles MP . Response rates in postal surveys of healthcare professionals between 1996 and 2005: an observational study. BMC Health Serv Res 2009;9:160. 10.1186/1472-6963-9-160 19751504 PMC2758861

[19] Hummers-Pradier E , Scheidt-Nave C , Martin H , Heinemann S , Kochen MM , Himmel W . Simply no time? Barriers to GPs’ participation in primary health care research. Fam Pract 2008;25(2):105–12. 10.1093/fampra/cmn015 18417465

[20] Herber OR , Schnepp W , Rieger MA . Recruitment rates and reasons for community physicians’ non-participation in an interdisciplinary intervention study on leg ulceration. BMC Med Res Methodol 2009;9:61. 10.1186/1471-2288-9-61 19682354 PMC2733138

[21] Nguyen TT , Love MB , Liang C , Fung LC , Nguyen T , Wong C , A pilot study of lay health worker outreach and colorectal cancer screening among Chinese Americans. J Cancer Educ 2010;25(3):405–12. 10.1007/s13187-010-0064-3 20204570 PMC2933803

[22] San Francisco Neighborhoods Socio-Economic Profiles: American Community Survey 2005-2009. San Francisco Planning Department, May 2011: 14-15. http://www.sf-planning.org/Modules/ShowDocument.aspx?documentid=8500. Accessed October 10, 2014.

[23] US Preventive Services Task Force. Screening for Colorectal Cancer: US Preventive Services Task Force Recommendation Statement. AHRQ Publication 08-05124-EF-3, October 2008. Agency for Healthcare Research and Quality, Rockville, MD. http://annals.org/article.aspx?articleid=743535. Accessed October 10, 2014.

[24] Kong H , Hsieh E . The social meanings of traditional Chinese medicine: elderly Chinese immigrants’ health practice in the United States. J Immigr Minor Health 2012;14:841–9. 10.1007/s10903-011-9558-2 22160808

[25] Sun WY , Basch CE , Wolf RL , Li XJ . Factors associated with colorectal cancer screening among Chinese-Americans. Prev Med 2004;39(2):323–9. 10.1016/j.ypmed.2004.04.029 15226041

[26] Maxwell AE , Crespi CM , Antonio CM , Lu P . Explaining disparities in colorectal cancer screening among five Asian ethnic groups: a population-based study in California. BMC Cancer 2010;10:214. 10.1186/1471-2407-10-214 20482868 PMC2888788

[27] Ramdass P , Petraro P , Via C , Shahrokni A , Nawaz H . Providers role in colonoscopy screening for colorectal cancer. Am J Health Behav 2014;38(2):234–44. 10.5993/AJHB.38.2.9 24629552

[28] Sung JJ , Choi SY , Chan FK , Ching JY , Lau JT , Griffiths S . Obstacles to colorectal cancer screening in Chinese: a study based on the health belief model. Am J Gastroenterol 2008;103(4):974–81. 10.1111/j.1572-0241.2007.01649.x 18047545

[29] Modiri A , Makipour K , Gomez J , Friedenberg F . Predictors of colorectal cancer testing using the California Health Inventory Survey. World J Gastroenterol 2013;19(8):1247–55. 10.3748/wjg.v19.i8.1247 23482920 PMC3587481

